# A Comparative Analysis of Wildland Fire Smoke PM_2.5_ Exposure Estimates Across California From 2008 to 2018

**DOI:** 10.1029/2025GH001575

**Published:** 2026-06-17

**Authors:** Rachel Connolly, Jenny T. Nguyen, Aron Walker, Joseph Wilkins, Yiqun Ma, Rosana Aguilera, Chen Chen, Alexander Gershunov, Joan A. Casey, Minghao Qiu, Danlu Zhang, Yang Liu, Tarik Benmarhnia, Michael Jerrett, Miriam E. Marlier

**Affiliations:** ^1^ Center for Healthy Climate Solutions Fielding School of Public Health, University of California Los Angeles Los Angeles CA USA; ^2^ Luskin Center for Innovation University of California Los Angeles Los Angeles CA USA; ^3^ Department of Environmental Health Sciences Fielding School of Public Health, University of California Los Angeles Los Angeles CA USA; ^4^ Scripps Institution of Oceanography University of California San Diego La Jolla CA USA; ^5^ Department of Earth, Environment, and Equity Howard University Washington DC USA; ^6^ Department of Environmental and Occupational Health University of Washington School of Public Health Seattle WA USA; ^7^ Department of Epidemiology University of Washington School of Public Health Seattle WA USA; ^8^ School of Marine and Atmospheric Sciences & Program in Public Health Stony Brook University Stony Brook NY USA; ^9^ Department of Biostatistics and Bioinformatics Rollins School of Public Health Emory University Atlanta GA USA; ^10^ Gangarosa Department of Environmental Health Rollins School of Public Health Emory University Atlanta GA USA

## Abstract

The development of smoke fine particulate matter (PM_2.5_) exposure surfaces for estimating air pollution trends and associated health effects has advanced considerably. Currently available smoke exposure products rely on various data sources and modeling techniques, as there is no gold standard method for modeling wildfire smoke PM_2.5_. This study compares multiple daily smoke PM_2.5_ data sets developed using diverse methodologies spanning 2008–2018. Incorporating metrics for short‐ and long‐term exposure, we compare four data sets at the census tract level in California: one using the U.S. Environmental Protection Agency’s chemical transport model (CTM), the Community Multiscale Air Quality Modeling System (CMAQ); two using statistical methods, also referred to as machine learning (ML) techniques; and one combining these approaches to develop an ML‐calibrated CTM‐based exposure surface. Our analysis highlights differences between the data sets in terms of long‐term exposure metrics, with the CTM data set estimating the highest concentrations overall, and considerable differences between estimates produced by the two ML models. An analysis of six case studies of large fires across the state finds that even data sets with similar inputs and methods produced estimates that varied several‐fold, with additional differences by region and over time. Our findings have important implications for quantifying smoke PM_2.5_ exposures for use in population health impact studies, which rely on exposure estimates to accurately estimate health burden from pollution exposure.

## Plain Language Summary

Over the last several decades, wildfires in the western U.S. have become more frequent and severe, exposing millions of people to toxic smoke pollution, including fine particulate matter (PM_2.5_). Several methods have emerged for modeling population exposure to this pollution. In this study, we compare four different smoke PM_2.5_ pollution models for California from 2008 to 2018. Our findings show significant differences in estimated smoke pollution exposure in both the short‐ and long‐term, even between similar modeling approaches. Many studies calculate the health burden of wildfire smoke exposure by comparing short‐ and long‐term health effects to short‐ and long‐term smoke pollution. Our findings suggest that health impact studies using smoke exposure estimates as a primary input may calculate differing health burden results based on the choice of smoke pollution data set.

## Key Points

- This is the first study comparing multiple daily smoke PM_2.5_ data sets over more than a decade in California with short‐ and long‐term exposure metrics
- We found distinct differences between estimates produced by atmospheric chemical transport models and machine learning models
- Results show the importance of quantifying added utility of each approach in developing fused surfaces to improve health burden estimates

## 1 Introduction

The frequency, severity, and intensity of wildfires have increased in the western U.S. due to a warming and drying climate, fuel build‐up, and human development in the wildland‐urban interface (Radeloff et al., 2023; Stephens et al., 2020; A. P. Williams et al., 2019). California has been one of the U.S. states most affected by fires, with annual burned area increasing fivefold from 1972 to 2018, largely due to increases in summertime forest burned area (A. P. Williams et al., 2019). Two wildfire regimes are dominant in California: fuel‐dominated summer forest fires and wind‐dominated autumn and (lately) winter coastal wildfires (MacDonald et al., 2023; A. P. Williams et al., 2019). Wind‐dominated wildfires occur in sloping coastal topography and are driven by downslope winds — Santa Ana in Southern California (Guzman‐Morales et al., 2016) and Diablo winds in Northern California (McClung & Mass, 2020). The same winds blow smoke towards densely populated coastal regions. With these two regimes active and enhanced by human activity, California's wildfire season spans summer, fall and winter, occasionally stretching into spring (MacDonald et al., 2023). Despite decades of air quality improvement achieved through reductions in other emissions sources, increased wildfire activity has reversed these gains, particularly in western U.S. states (Burke et al., 2023; McClure & Jaffe, 2018).

Wildland fires, which include wildfires, prescribed fires, and managed wildfires, emit multiple trace gas and aerosol species into the atmosphere. Fine particulate matter (PM_2.5_) is one of the most studied exposure indicators in the environmental health field, with people exposed from many sources, both natural and anthropogenic, including wildland fires. Total PM_2.5_ exposures can be estimated with ground monitors (physical measurements), satellites, and/or atmospheric models (Diao et al., 2019). A core challenge, however, with estimating the contribution of wildland fires to ambient PM_2.5_ concentrations, hereafter referred to as “smoke PM_2.5_,” is that measurements capture only total PM_2.5_ and do not distinguish fire‐attributable particles from other sources. As a result, there is no physical measurement that can directly quantify smoke PM_2.5_ and thus be used as a gold standard. Several approaches, however, such as chemical transport models (CTMs) and statistical models (as described in (Jaffe et al., 2020)), can be used to estimate smoke PM_2.5_. As these methods rapidly evolve, it is critical to continuously compare the results of each approach and how differences impact subsequent analyses.

The spatial and temporal distribution of smoke PM_2.5_ exposure has important implications for public health, and in particular for the health of community members who are more vulnerable to adverse health outcomes, such as older adults, young children, pregnant women, and individuals with pre‐existing health conditions (Cascio, 2018). Epidemiological studies have found considerable evidence of the adverse health impacts of both urban and wildfire‐specific PM_2.5_ (Black et al., 2017; Cascio, 2018; Liu et al., 2015; Pope & Dockery, 2006; Reid et al., 2016), with growing epidemiological and toxicological evidence that wildfire smoke may be a particularly toxic form of PM_2.5_ (Aguilera et al., 2021; Lei et al., 2024; Wegesser et al., 2009; V. A. Williams et al., 2024). While the vast majority of previous studies focus on short‐term exposure and health effects, recent studies highlight a need to address long‐term and repeated exposure to smoke PM_2.5_ (Casey et al., 2024; Sacks et al., 2025). It is thus critical to assess how multiple exposure modeling approaches compare across a variety of short‐ and long‐term exposure metrics.

CTMs simulate the influence of wildland fire emissions on smoke PM_2.5_ and other pollutants without the use of observational air pollution monitoring data as a model input. CTMs rely on gridded emissions estimates from wildland fires and other sources and use simulations of the chemical and physical processes in the atmosphere to model the spatial distribution of pollution (Kelp et al., 2023; Schollaert et al., 2024; J. L. Wilkins et al., 2018). Several CTM smoke PM_2.5_ output data sets are publicly available (Qiu, 2024; J. Wilkins & Connolly, 2024), including those used in Connolly et al. (2024) and Qiu et al. (2024). CTMs have multiple advantages. First, they can simulate smoke PM_2.5_ concentrations in areas located far from monitors or when satellites have an uncertain view of surface concentrations, for example, due to cloud coverage. They can also estimate contributions from specific sources of smoke PM_2.5_, such as wildfires, prescribed fires, or agricultural burns, by running a series of simulations in which different emissions sources are included in the modeling framework (Schollaert et al., 2024). Finally, CTMs can also be used to estimate the influence of future changes in climate or wildland fire management (Liu et al., 2016; Neumann et al., 2021). Despite these advantages, however, CTMs are computationally intensive and rely on uncertain inputs of fire emissions, injection heights, and other processes that can influence modeled concentrations (Carter et al., 2020; Feng et al., 2023; T. Liu et al., 2020). Particularly, choice of emissions source and associated uncertainties can result in substantial differences between CTM model outputs (Jaffe et al., 2020). Additionally, CTM smoke PM_2.5_ concentrations have been found to overestimate concentrations in some cases (Qiu et al., 2024).

Statistical or machine learning (ML) approaches for estimation of smoke PM_2.5_ typically combine satellite observations of smoke plumes and/or active fire detection, ground air pollution monitoring data, and other surface properties such as meteorology and land cover to estimate concentrations in places without ground monitors (Aguilera et al., 2023; Childs et al., 2022). Two publicly available data sets based on this approach use varying methodologies, though both use the National Oceanic and Atmospheric Administration's Hazard Mapping System (HMS) smoke estimates to identify days with ground‐level smoke (Aguilera et al., 2023; Childs et al., 2022). HMS, however, only represents smoke levels in the atmospheric column, not necessarily at the surface, and researchers have identified considerable biases in using HMS data as a tool for estimating ground‐level smoke (T. Liu et al., 2024). Using an ML approach, Aguilera et al. (2023) developed a daily smoke PM_2.5_ data set for California at the ZIP code level from 2006 to 2020. Childs et al. (2022) also applied a ML approach, estimating daily smoke PM_2.5_ for the contiguous U.S. at a 10‐km scale from 2006 to 2020. Another study compared two ML total PM_2.5_ products (Di et al., 2019; Reid et al., 2021) during wildfire events, finding that each data set underestimated PM_2.5_ concentrations on high pollution days, although these data sets did not explicitly estimate the smoke PM_2.5_ fraction (Considine et al., 2023). Another recently published data set (Zhang et al., 2023) took a multistage approach with a CTM‐based modeling framework, using various satellite, land‐use, and ground‐station monitoring inputs and then incorporating ML approaches to downscale estimates to a 1‐km grid for the period of 2007–2018 across the contiguous U.S.

Reducing uncertainty in smoke PM_2.5_ exposure assessment is critical for increasing the accuracy of epidemiological studies investigating the health effects of wildfire smoke and the downstream health impact estimates that apply developed concentration‐response estimates to exposure surfaces to estimate the burden of air pollution exposure (Connolly et al., 2024; Fann et al., 2018). Including unrealistically high smoke PM_2.5_ concentrations from a CTM simulation (J. L. Wilkins et al., 2018) increased mortality burden assessments by ∼6% (Connolly et al., 2024). Another study compared population exposure estimates from a CTM model, surface PM_2.5_ monitors, and satellite aerosol optical depth, also applying a data fusion approach using ridge regression techniques, finding that blending techniques can offer substantially improved estimates, but only when availability of monitors is limited (Lassman et al., 2017). In a companion study, authors found that the strength and significance of association with cardiopulmonary hospital admissions varied by PM_2.5_ estimation method during a severe wildfire season in Washington State (Gan et al., 2017). Authors of a Colorado‐based analysis of cardiopulmonary health effects from wildfire smoke also explicitly noted challenges in directly comparing results across studies that applied different exposure assessment approaches and evaluated differing health outcome categories (Magzamen et al., 2021), citing an order of magnitude difference between outcomes for studies utilizing different PM_2.5_ estimation methods (Alman et al., 2016; Stowell et al., 2019). Recent work for the 2020 fire season compared two CTMs with a ML data set and found that the CTM simulations overestimated smoke PM_2.5_ concentrations during extreme events (Qiu et al., 2024). The analysis also demonstrated that using different exposure data sets to (a) develop the concentration‐response function, as well as (b) estimate downstream attributable health impacts both result in substantive differences in estimated health burden, though all along the same order of magnitude (Qiu et al., 2024). The extent to which the divergence between modeling approaches varies by fire type and over time is largely unknown.

In this study, we compare four daily smoke PM_2.5_ data sets derived using different modeling approaches over an 11‐year period (2008–2018) at the census tract level in California, building on a recent review paper comparing data products that provided a limited comparison between three of the same data sets for two years (Orr et al., 2025). These four data products were selected for our analysis based on their availability for the fire‐prone region of California, overlapping time periods during which the state experienced several high‐fire and low‐fire years, and utilization of diverse methodologies particularly relevant for comparative analysis. We first compare the smoke PM_2.5_ products across several long‐term smoke exposure metrics of frequency, duration, and intensity, and then analyze six case studies of large fire events in different seasons and years. The data sets include one CTM (J. L. Wilkins et al., 2018), two data sets using ML approaches (Aguilera et al., 2023; Childs et al., 2022), and one fusion data set combining a CTM with ML techniques (Zhang et al., 2023). While we use the term CTM here to distinguish between model types, we also note that CTM outputs can vary significantly depending on emissions data and other inputs; however, the smoke PM_2.5_ concentrations from the CTM used in the Zhang estimates (Koman et al., 2019) are similar to the Wilkins estimates with respect to overall approach and descriptive statistics (Connolly et al., 2024). Although we anticipate substantial similarities between the two ML data sets, as they have similar methodologies and inputs, the comparison across different metrics as well as case studies will enable us to characterize potential differences and compare within‐method variation to cross‐method variation. Moreover, it is important to understand how the calibrated CTM data set compares to both solely CTM and ML data sets and what potential opportunities it presents for smoke PM_2.5_ estimation. This analysis will provide novel insights into the nature of the differences between available smoke PM_2.5_ products.

## 2 Materials and Methods

### 2.1 Smoke PM_2.5_ Data Sets

We included four smoke PM_2.5_ data sets in our analysis, including one CTM, two ML data sets, and one fusion data set combining a CTM with ML techniques (Table S1 in Supporting Information S1). All four data sets provided isolated smoke PM_2.5_ concentrations in μg/m^3^ at a daily scale, though they applied different methodologies and developed data at different spatial resolutions. We limited our analysis to California from 2008 to 2018 as this is the maximum spatial and temporal domain common to all four data sets. We conducted our entire analysis at the census tract level, as the two ML data sets (Aguilera et al., 2023; Childs et al., 2022) were readily available at that spatial resolution and it is also relevant for population health and vulnerability analyses. Average smoke PM_2.5_ levels over the 11‐year period at the census tract level are shown in Figure S1 in Supporting Information S1. Population‐weighting approaches were used for all data aggregation at the tract level.

We used CTM data modeled through the U.S. Environmental Protection Agency’s (EPA) Community Multiscale Air Quality Modeling System (CMAQ) tool, v. 5.0.1–5.3 (see (Connolly et al., 2024; Jung et al., 2024) for details on model specifications). The CMAQ outputs included daily modeled smoke PM_2.5_ concentrations (including wildfires and prescribed burns) for 2008–2018 for the state of California at a 12‐km grid spatial resolution. We set all modeled smoke PM_2.5_ concentrations <0 μg/m^3^ to 0 μg/m^3^, which accounts for approximately 11.7% of gridded fire‐only observations. This is due to nonlinearities in CTM processing resulting in similar (near‐zero) values for all‐sources and non‐fire concentrations on non‐smoke days, considering non‐fire estimates were subtracted from all‐sources concentrations to estimate fire‐only smoke PM_2.5_ (see (Connolly et al., 2024)); that is, 97% of negative values are between 0 and ‒0.05. We aggregated these data to the population‐weighted census tract centroid. Validation for 2008–2012 is available in (J. L. Wilkins et al., 2018). This data set is hereafter referred to as the “Wilkins” data set.

The second data set is a ML data set with daily smoke PM_2.5_ estimates for the contiguous U.S. on a 10‐km grid, also available at the census tract level as an area‐ and population‐weighted average of intersecting grid cells, derived from the native grid (Childs et al., 2022), hereafter referred to as the “Childs” data set. The authors estimated smoke PM_2.5_ concentrations through the following approach: (a) developing a binary classification for smoky days using satellite imagery smoke plume classification and modeled air trajectories (the latter as needed to account for cloud coverage), (b) estimating smoke PM_2.5_ based on PM_2.5_ anomalies at U.S. EPA ground stations on the classified smoky days, measured as differences from monthly median PM_2.5_ on non‐smoke days, and (c) training a statistical model to predict those concentrations. The authors reported *R* ^2^ = 0.67 at monitoring stations not used for training, and model performance was higher in locations with higher smoke concentrations and more variability in concentrations; it was reduced in regions with higher variance in non‐smoke concentrations. These data were readily available at the tract level and were not processed or aggregated prior to the analysis.

The third data set is a ML data set providing daily total and smoke PM_2.5_ estimates for California at the census tract population‐weighted centroid level (data are also publicly available at the ZIP code‐level) (Aguilera et al., 2023; Casey et al., 2024), hereafter referred to as the “Aguilera” data set. The authors followed a similar approach to (Childs et al., 2022), though the authors estimated all‐source PM_2.5_ for all days and census tracts first, followed by non‐smoke PM_2.5_ with another set of models. The authors estimated smoke PM_2.5_ concentrations through the following approach: (a) developing a binary classification for smoky days using satellite imagery smoke plume classification, (b) estimating daily, all sources PM_2.5_ for each census tract population‐weighted centroid by training an ensemble model, (c) isolating non‐smoky days in the data set and imputing values of non‐smoke PM_2.5_ on smoky days using a random forest approach, and (d) subtracting non‐smoke PM_2.5_ from all sources PM_2.5_. Explanatory variables for the ML model, including the binary plume classification, were taken at the population‐weighted centroid of each tract. The authors reported *R* ^2^ = 0.83 for total PM_2.5_ at U.S. EPA Air Quality System (AQS) monitoring sites and 0.78 for the hold‐out data set not used in training. These data were readily available at the tract level and were not processed or aggregated prior to the analysis.

The fourth data set used in our study combined CTM and ML approaches. The authors developed 1‐km grid‐level PM_2.5_ estimations derived from two random forest models, with the CMAQ CTM as one of the primary data inputs (Zhang et al., 2023), hereafter referred to as the “Zhang” data set. The CMAQ model isolates smoke PM_2.5_, as mentioned previously. The model for smoke‐impacted regions (by day), determined using HMS plume polygons and ratios of CMAQ smoke PM_2.5_ as compared to total PM_2.5_ over a tested threshold, provided predictions for total PM_2.5_, while the model for non‐smoke regions estimated background PM_2.5_, with total PM_2.5_ assumed to equal background PM_2.5_ in non‐smoke regions. The daily smoke PM_2.5_ concentration for each grid cell in smoke‐impacted regions was then calculated as the difference between the predicted total PM_2.5_ and the predicted background PM_2.5_. The authors reported an overall *R* ^2^ = 0.75 for their smoke model when compared to AQS monitoring sites. We aggregated these data to the population‐weighted census tract centroid. The CMAQ outputs used to develop the Zhang estimates were not from the exact same model runs as Wilkins but use similar inputs and modeling approach, including use of the BlueSky framework and SMARTFIRE2 to develop emissions (see (Connolly et al., 2024; Wilkins et al., 2018)).

### 2.2 Comparison and Validation of Smoke PM_2.5_ Concentrations

We developed descriptive statistics and correlations for smoke PM_2.5_ comparison at the census tract level. Using commonly employed model validation statistics (Appel et al., 2011; Wilkins et al., 2018) including correlation coefficient, coefficient of determination, and metrics of bias and error, we also compared all four data sets to ground monitoring data, the latter of which notably do not include an attributed concentration of smoke PM_2.5_ for direct comparison (they just include total PM_2.5_). Many of the permanent U.S. EPA ground station monitors were used in training for the ML data sets and have sparse spatial coverage in rural, fire‐prone areas. Instead, we used temporary ground monitoring data deployed during wildfire events by the U.S. Forest Service (USFS). The monitors are typically deployed in locations downwind of fires where there are concerns about population impacts (Considine et al., 2023) and present meaningful additional information beyond permanent monitors with respect to characterizing human exposures (Jaffe et al., 2020). For this supplemental validation analysis, we used cleaned data provided by and described in Considine et al.; data processing included the removal of all observations higher than 1,000 μg/m^3^ (Considine et al., 2023). Figure S2 in Supporting Information S1 shows the location of the USFS temporary monitors in the complete Considine data set along with U.S. EPA AQS ground station PM_2.5_ monitoring locations. We also include both linear (Pearson) and nonparametric (Spearman) correlations between each data set, calculated for daily observations; using the Spearman method reduces the potential impact of outliers on the correlation coefficient. However, this comparison analysis is limited by several factors, including a small number of paired census tract–days; the use of total ambient PM_2.5_ rather than isolated smoke PM_2.5_; and higher measurement uncertainty relative to regulatory‐grade instruments. Therefore, while we include this analysis here as we aim to provide an exploratory assessment, results should be interpreted cautiously.

As wildfires have become more common and severe in California, it is pertinent to evaluate long‐term exposures, as populations are increasingly exposed to smoke through repeated, episodic events of varying duration and intensity (Sacks et al., 2025). Casey et al. (2024) recently proposed a conceptual model for characterizing long‐term smoke exposures across multiple dimensions, including frequency, duration, and intensity, explicitly accounting for the distinctive temporal patterns of wildfires. Here, we adapt this framework and compare the exposures quantified by each data set with respect to four long‐term smoke PM_2.5_ metrics. These include: (a) the number of weeks each year for which mean smoke PM_2.5_ concentrations exceeded 5 μg/m^3^; (b) the number of days each year for which wildfire PM_2.5_ concentrations were >1 μg/m^3^; (c) the number of smoke waves each year, defined as instances of ≥2 consecutive days with >15 μg/m^3^ wildfire PM_2.5_; and (d) the mean annual wildfire PM_2.5_ concentration. Additional methodological details on the calculation of these metrics are included in the supplement. Finally, we also used paired *t*‐tests to compare census tract‐level exposure estimates across all pairs of data sets for each metric, using values aggregated over the study period.

### 2.3 Wildfire Case Studies

To evaluate the performance of each data set under varying conditions, seasons, and time periods, we analyze six case studies spanning the 2008–2018 period, selected to include large fires that occurred during different seasons, across a gradient of rural to urban areas, and in different ecoregions (Table 1, Figure S3 in Supporting Information S1). We compare across frequency, duration, and intensity as appropriate. For each case study, we included all census tracts within the county or counties in which the fire burned, aiming to capture the most smoke‐affected areas near the fire perimeter. We compare smoke PM_2.5_ estimates using three complementary temporal periods: the entire fire duration; the week of each fire with the highest emissions, referred to as “peak week; ” and the first 2 weeks of each fire, referred to as the “fire start.” The latter two timeframes align for several of the case studies, but not for all; specifically, the Basin Complex and Rough Fire have peak weeks beyond the fire start.

**Table 1:** Description of Case Studies

Fire or complex name	Month/Year ignited	Fire duration^a^	Acres burned	Affected counties^b^
Basin Complex	June 2008	6/21/2008–7/27/2008	162,818	Monterey
Rough Fire	July 2015	7/31/2015–12/1/2015	151,623	Fresno
Wine Country Fires—Tubbs, Atlas, and Nuns	October 2017	10/8/2017–10/31/2017	133,004 (combined)	Napa, Sonoma
Thomas Fire	December 2017	12/4/2017–1/12/2018	281,893	Ventura, Santa Barbara
Mendocino Complex	July 2018	7/27/2018–9/27/2018	459,123	Mendocino, Lake, Colusa, Glenn
Woolsey Fire	November 2018	11/8/2018–11/22/2018	96,949	Los Angeles, Ventura

^a^ From ignition to containment according to CAL FIRE Redbooks (CAL FIRE, 2024).

^b^ Counties intersecting with the fire perimeter.

Importantly, this approach is limited in that it does not necessarily incorporate all counties downwind and affected by a given smoke event, as it is not possible to isolate impacts of specific fires using the available data sets. In addition, smoke concentrations during the case study period may reflect contributions from fires other than the focal events. For example, the Carr Fire in Shasta was burning in the same period as the Mendocino Complex and likely contributed to smoke observed in counties where the Mendocino Complex was actively burning. With these limitations in mind, the objectives of this analysis are (a) to provide illustrative examples that compare the behavior of the four data sets during periods and in regions with large fires, and (b) to present an internally consistent comparison in the areas expected to experience the most immediate and consistently elevated smoke impacts.

## 3 Results

### 3.1 Summary Statistics and Validation

**Figure 1:**
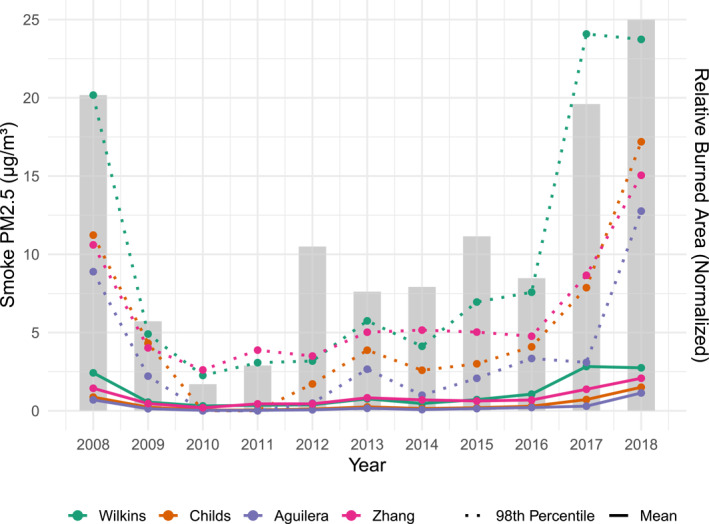
Smoke PM_2.5_ summary statistics (mean and 98th percentile of all census tract estimates) by year (μg/m^3^). Note: Acres burned were extracted from CAL FIRE Redbooks for each year (https://www.fire.ca.gov) and are depicted (by the Gy bars) relative to the year with the highest burned area, 2018. Total acres burned from 2008 to 2018 in order of year: 1,593,690; 451,969; 134,462; 228,599; 829,224; 601,635; 625,540; 880,899; 669,534; 1,548,429; and 1,975,086.

Figure 1 presents the mean and 98th percentile of smoke PM_2.5_ for each year of the data set included in our analysis over the entire state, alongside relative burned area.

Within each data set, higher mean smoke PM_2.5_ estimates track years with the most burned area, particularly 2008, 2017, and 2018, with the exception of the Aguilera data set for 2017. Despite more than 1.5 million acres burned in 2017, the Aguilera data has a mean smoke PM_2.5_ concentration of 0.30 μg/m^3^, which is similar in magnitude to other years with considerably less fire activity; this may reflect more fire activity in rural areas further from the population‐weighted census tract centroids used to develop the Aguilera estimates. Across the four data sets, the CTM estimates from Wilkins are greater than the ML data sets on an annual level, with consistently higher mean values for each year that are more than double the Childs and Aguilera data sets for most years. Overall, the Zhang estimates are the closest to the Wilkins estimates, with higher mean concentrations for four of the low‐moderate fire years (2011–2014), but lower estimates for the other years. For the highest fire year in terms of burned area—2018—the mean estimate for Zhang is 2.10 μg/m^3^ compared to 2.75 μg/m^3^ for Wilkins, followed by 1.51 μg/m^3^ for Childs and 1.14 μg/m^3^ for Aguilera.

The 98th percentile estimates follow similar patterns between the data sets. For example, in 2018, 98th percentiles range from 12.8 μg/m^3^ for the Aguilera data set to 23.8 μg/m^3^ for the Wilkins data set. Additionally, while the ratio of 98th percentile to mean is consistent for the Wilkins and Zhang data sets (which both employed CMAQ), the ratio between the two varies more inconsistently for the ML data sets. In the low fire years, the 98th percentiles for the ML data sets are often lower than the mean, likely due to the large number of 0 μg/m^3^ estimates for non‐smoke days accompanied by several outlier values.

Figure S4 in Supporting Information S1 includes linear (Pearson) and nonparametric (Spearman) correlations between each data set for the fires season (June–October), based on daily observations. We found considerable differences between the two methods in most cases. Unsurprisingly, we find that the two ML data sets (Childs and Aguilera), which used similar data inputs and methodology in development of their exposure estimates, are highly correlated for all years. While all correlations are positive, correlations comparing the Wilkins and Zhang data sets to each of the Childs and Aguilera data sets are considerably lower (overall lowest for Wilkins compared to the two ML data sets). The nonparametric correlations are quite high between the Wilkins and Zhang data sets, ranging from 0.55 in 2010 (a low fire year) to 0.79 in 2018 (a high fire year). The Zhang data set uses CMAQ as a primary input, so as with comparing the two ML data sets, higher correlations in this case are expected, particularly as the CMAQ models in both cases used the BlueSky emissions framework (Koman et al., 2019; J. L. Wilkins et al., 2018; Zhang et al., 2023), and summary statistics were comparable across the models (Connolly et al., 2024).

The validation with the USFS temporary monitors (Table S2 in Supporting Information S1) is limited by a lower number of paired census tract‐days and measurements of total PM_2.5_ rather than isolated smoke PM_2.5_. We find higher mean concentrations for the monitoring data than for all modeled data sets we evaluated for the dates and census tracts with paired observations available. Bias estimates demonstrate that all four modeled data sets predict lower smoke PM_2.5_ concentrations than the monitoring data, with mean bias in high fire years (2008, 2017, 2018) ranging from −4.12 to −8.95 μg/m^3^ for Wilkins, and greater than −10 μg/m^3^ for those years in the other three data sets, with the exception of −9.05 μg/m^3^ for Zhang in 2008. Lower bias estimates are expected, given the temporary monitors will include all other sources of PM_2.5_ along with smoke; however, the expected magnitude of this difference is challenging to quantify, and results should be interpreted accordingly. Root mean square error for the Wilkins data set is consistently the highest, though with the lowest mean bias; this indicates that the data set may have large individual errors, such as those due to outliers, rather than systematic underestimation. However, these results should again be interpreted within the context that temporary monitors capture concentrations at limited locations and over restricted time periods. Additionally, they are placed in a specific location that is different from the population‐weighted centroids used for aggregating three out of our four data sets (Childs is both area‐ and population‐weighted), which may be particularly consequential in larger, more rural tracts. Finally, there is less certainty in temporary monitoring instrument measurements (Schweizer et al., 2016), as they are not stationary equipment and do not meet regulatory compliance standards (i.e., Federal Reference or Equivalent Methods [FRM/FEM]).

### 3.2 Spatial and Temporal Comparison of Long‐Term Exposure Metrics

**Figure 2:**
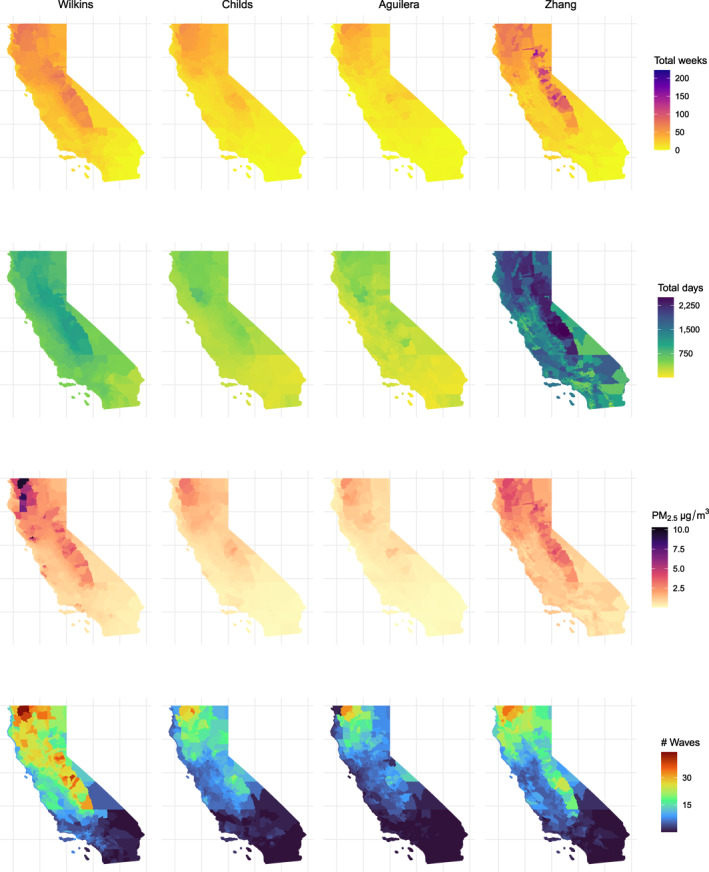
Spatial metrics of intensity, frequency, and duration for smoke PM_2.5_ in California, 2008–2018. Number of weeks with smoke PM_2.5_ exceeding 5 μg/m^3^ (top row), number of days with smoke PM_2.5_ exceeding 1 μg/m^3^, indicating nonzero smoke exposure (second row), mean smoke PM_2.5_ (third row), number of smoke waves (fourth row). Note: Smoke waves represent the number of instances of ≥2 consecutive days with >15 μg/m^3^ wildfire PM_2.5_

Here, we present a comparison of the four data sets with respect to metrics of duration, intensity, and frequency, as presented in (Casey et al., 2024). Figure 2 shows the spatial distribution, Figure S5 in Supporting Information S1 shows the temporal trends over the 11‐year period, and Figure S6 in Supporting Information S1 shows the census tract frequency distribution of estimates for each metric. Overall, the Wilkins data set presented the highest estimates, followed by Zhang, Childs, and Aguilera.

The Wilkins data set had the most weekly smoke PM_2.5_ exceeding 5 μg/m^3^ over the 11‐year period, with an average of 4.7% of tract‐weeks from 2008 to 2018, followed by 2.1% for Zhang, 1.3% for Childs, and 0.8% for Aguilera. Figure 2 demonstrates that most of those weeks were concentrated in northern and central California, with striking similarities between the Wilkins and Zhang data sets, as well as between the Childs and Aguilera data sets. A comparison of total days exceeding a low threshold for smoke PM_2.5_ exposure is slightly more challenging with these four data sets, as the Childs and Aguilera data sets have many days with concentrations of 0 μg/m^3^ due to the use of smoke plumes to characterize binary smoke and non‐smoke days, whereas CTM methods result in many low‐level concentrations near, but above, 0 μg/m^3^. Therefore, we use a threshold of 1 μg/m^3^ to identify smoke‐exposed tract‐days (we tested various thresholds, and trends are similar if 2 μg/m^3^ is applied). Zhang's combination of ML and CTM approaches resulted in a high number of days with at least low‐level exposures (>1 μg/m^3^), and the distribution is particularly varied for tract‐days compared to the other three data sets (Figure S6 in Supporting Information S1). For Zhang, we observe a census tract average of 23% of all days from 2008 to 2018 exceeding the threshold, followed by Wilkins with pure CTM methods at 16%, and then Childs with 5.6% and Aguilera with 3.7%.

Average smoke PM_2.5_ concentrations over the 11‐year period are highest for Wilkins (Figure 2), with hot spots again in northern California and throughout the Central Valley, ranging from 0.29 μg/m^3^ for the least exposed tract in San Bernardino County to 10.3 μg/m^3^ for the most exposed tract in Sonoma County. For Childs, Aguilera, and Zhang, the most exposed tract was in Siskiyou County, with period‐wide average concentrations of 3.1, 2.4, and 4.2 μg/m^3^, respectively. Wilkins also had a high estimate (9.4 μg/m^3^) for that tract.

For smoke waves, the largest number of ≥2 days waves (with concentrations greater than 15 μg/m^3^) was observed in the northernmost part of the state for all data sets (Figure 2). Wilkins demonstrated the widest distribution in frequency of smoke wave counts across tracts (Figure S6 in Supporting Information S1).

We observe both inter‐metric and inter‐dataset variation in temporal patterns (Figure S5 in Supporting Information S1), supplementing and echoing the spatial patterns demonstrated in Figure 2. The high‐fire years of 2008, 2017, and 2018 dominate all four of the exposure metrics.

A supplemental statistical analysis using paired *t*‐test comparisons between data sets demonstrated statistically significant differences for all metrics when comparing census tract–level estimates aggregated over the full study period, which is unsurprising given the large number of census tracts (and associated statistical power). Mean differences (Figure S7 in Supporting Information S1) and effect sizes (Cohen's d, Figure S8 in Supporting Information S1) emphasize the variations described previously, with the highest effect sizes—quantifying by how many standard deviations the group means differ—for differences between Wilkins and Childs, and Wilkins and Aguilera.

### 3.3 Case Studies

**Figure 3:**
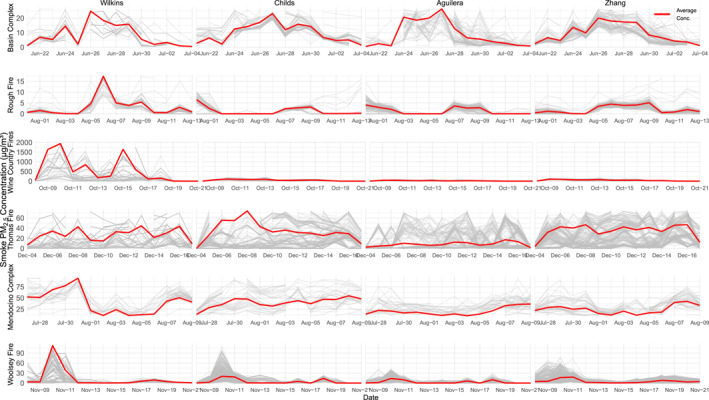
Average (red) and tract‐level (gray) smoke PM_2.5_ concentrations for the first 2 weeks of each case study for each data set. Notes: Gray lines reflect individual census tract estimates, and red lines the overall average for all tracts in all counties containing any portion of the fire perimeter. Extreme observations beyond each plot's *y*‐axis are not included.

We selected six illustrative case study fires from high‐burn years across different seasons (Table 1), including summertime lightning‐ignited fires and catastrophic, wind‐driven coastal fires—typically human‐caused due to the absence of natural ignition sources, often from power lines. Figure S9 in Supporting Information S1 shows average smoke PM_2.5_ concentration throughout the fire duration (from ignition to CAL FIRE containment), and Table 2 summarizes smoke PM_2.5_ concentrations for (a) the entire fire duration and (b) the peak week of each fire, based on PM_2.5_ emissions data from the Wildfire Burn Severity and Emissions Inventory (WBSE) (Xu et al., 2022a, Xu et al., 2022b), as well as the number of smoke waves during that period. Figure 3 shows the daily mean and range of smoke concentrations for the first 2 weeks of each fire, or the fire start, including all census tracts in the entire affected county or counties overlapping with the fire perimeter. This case study analysis expands our understanding of the distinctions between data sets beyond the state‐level results presented in the previous section. Here, we present results by case study, listed in chronological order.

**Table 2:** Case Study Results Summary

Case study	Mean smoke PM_2.5_ for tracts in affected counties, entire fire duration (μg/m^3^)^a^				Mean smoke PM_2.5_ for tracts in affected counties, peak week of the fire (μg/m^3^)^b^			
	Wilkins	Childs	Aguilera	Zhang	Wilkins	Childs	Aguilera	Zhang
Basin Complex	10.8	8.30	5.56	9.04	21.6	10.2	5.15	12.6
Rough Fire	4.34	1.16	1.12	2.51	25.4	11.4	10.6	12.7
Wine Country Fires	330	26.7	9.31	25.3	998	73.3	26.4	69.0
Thomas Fire	10.9	12.4	2.96	13.4	27.0	46.1	7.83	38.1
Mendocino Complex	16.9	16.4	9.51	11.6	36.0	40.5	14.1	18.2
Woolsey Fire	12.2	4.12	2.79	5.04	22.4	6.09	3.62	6.27

^a^ As described in Section 2.3, we define affected counties as those intersecting with the fire perimeter.

^b^ Peak week for each fire determined based on rolling averages of total PM_2.5_ emissions from the Wildfire Burn Severity and Emissions Inventory (WBSE).

The Basin Complex broke out in the Ventana Wilderness near Big Sur in Monterey County after ignition by a lightning strike in the summer of 2008 (CAL FIRE, 2024). Unlike most fires analyzed, for the fire start the Basin Complex follows more consistent patterns across data sets with respect to the timing of daily average concentration peaks across the county (Figure 3). The Wilkins estimates are higher for the entire fire duration by several μg/m^3^, nearly twice the magnitude of Aguilera estimates and 20% larger than Zhang estimates (Table 2). During the peak week of the fire, the average concentration for Wilkins is more than twice Childs and four times Aguilera for that 7‐day period; however, this discrepancy is notably not captured in Figure 3 because the peak week of emissions began in July and was beyond the fire start dates shown in Figure 3. Interestingly, the Aguilera estimates for the peak week (Table 2) are half the Childs concentrations. The Zhang estimates are slightly higher than the Childs estimates. The spatial trends of the Wilkins and Zhang distributions are quite similar (Figure S9 in Supporting Information S1); Childs differs in that it does not show as many census tracts with hot spots but still presents a similar spatial trend, and the Aguilera estimates, again, are quite visibly low and do not demonstrate the same spatial hotspots.

The Rough Fire was the largest fire in the state in 2015, ignited by a lightning strike in Fresno County (CAL FIRE, 2024). Much of the burning occurred in the Sierra and Sequoia National Forests, though several structures were destroyed. For this fire, the Aguilera and Childs estimates were within 1 μg/m^3^ for both the overall average and the peak week estimates (Table 2). The Zhang estimates are closer in magnitude to the ML estimates, with the Wilkins concentrations again much higher—approximately double or more than double of all the three other data sets for the peak week average concentrations. Interestingly, the Wilkins data set shows a distinct peak on one of the days during the fire start period that is not reflected in any of the other data sets (Figure 3). Figure S9 in Supporting Information S1 demonstrates similar spatial patterns for the four data sets, though the significantly higher concentrations for Wilkins, as seen in Table 2, are apparent.

The three Wine Country Fires included in our case study started in October 2017 and ultimately resulted in the deaths of more than 20 people and destruction of thousands of structures (CAL FIRE, 2024). These three fires were all ignited by various electrical system failures (several involving trees coming into contact with power lines). The Wilkins estimates during this fire event are extremely high, with an average of 330 μg/m^3^ for tracts in affected counties. This average is driven by the peak week of the fire, which reached 998 μg/m^3^ in the Wilkins estimates (Table 2, Figure 3). Because the Wilkins estimates are substantially higher (Figure 3), Figure S10 in Supporting Information S1 also presents a stratified version of the Wine Country Fires analysis that excludes Wilkins, allowing the remaining three data sets to be shown on an appropriate scale. Table 2 and Figure S10 in Supporting Information S1 reveal that concentrations were also particularly high for Childs and Zhang during this fire event, with peak week concentrations of approximately 70 μg/m^3^. However, this is more than an order of magnitude lower than the CTM estimates in the Wilkins data sets. Figure S9 in Supporting Information S1 demonstrates similar spatial trends for all four data sets despite differences in magnitude. The same high concentrations are not present in the Zhang estimates, which also used a CTM.

The Thomas Fire was a late‐season Santa Ana wind‐driven coastal wildfire that broke out in December 2017 in Ventura and Santa Barbara, ultimately determined to be ignited by power lines (CAL FIRE, 2024; KCBX Central Coast, 2019; Raphelson, 2017). This fire demonstrates some different trends compared to the other case studies. Wilkins' overall average is lower than both Childs and Zhang, as is the peak week estimate (Table 2). Also, during the entire duration and peak week of the Thomas fire, the Aguilera average is four and nearly six times lower than the Childs average, respectively. Figure 3 further indicates a drastic difference between the two ML data sets for this fire, mirroring evidence from Table 2.

The Mendocino Complex was a human‐caused fire in the summer of 2018 in northern California (CAL FIRE, 2024). For this case study, the Wilkins estimates are similar to Childs for the entire fire duration, with average concentrations of 16.9 μg/m^3^ and 16.4 μg/m^3^ for Wilkins and Childs, respectively (Table 2). Estimates are slightly lower for Wilkins than Childs for the peak week, and Aguilera and Zhang estimates are considerably lower (Table 2). With respect to spatial dynamics, Figure S9 in Supporting Information S1 shows that smoke PM_2.5_ is more widely spread throughout the affected counties for Wilkins, while Childs shows smoke PM_2.5_ as more centralized in one area. Furthermore, Wilkins shows a large peak a few days after the start of the fire, which is missing from the other three data sets when considering the average (red line), though the range (gray lines) indicates that some census tracts reached similar levels (Figure 3).

The Woolsey Fire—a coastal Santa Ana wind‐driven wildfire—ignited in November 2018 in southern California, caused by power lines (State of California Department of Justice: Office of the Attorney General, 2021), burning more than 1,600 structures (CAL FIRE, 2024). It was another particularly catastrophic human‐caused coastal wildfire. This fire occurred only a few months after the Mendocino Complex but in a different region, and we see different trends in spatial smoke distribution in Figure S9 in Supporting Information S1, with a significant hot spot rather than widespread air quality impacts. Similar to the Mendocino Complex, the Wilkins data set shows a large peak on one day near the start of the fire, which is not consistent with the other data sets (Figure 3). Additionally, the Wilkins data set stands out with a peak week estimate of 22.4 μg/m^3^, with the others considerably lower, ranging from 3.62 to 6.27 μg/m^3^ (Table 2).

The data set with the highest concentration for the entire fire and during the peak week of emissions often varies by the fire event (Table 2). However, trends remain consistent from the entire fire duration to the peak week average for most case studies, with some minor variations. The differences between ML data sets indicate that even when using similar data inputs and methodological approaches, results may vary significantly, with stark differences for some case studies, such as the Thomas Fire. Figure 3 shows that for some fires, the timing of peak concentrations within the first 2 weeks varies significantly depending on whether CTM or ML methods were used, and for others, temporal patterns are more consistent. Interestingly, the Zhang data set follows Wilkins' trends for some fire events but aligns with the ML data sets for others. As also demonstrated in Sections 3.1 and 3.2, Aguilera's estimates are notably lower than other data sets for all fire events. The Aguilera estimates are between 1.5 and >4 times lower than the other data sets for all case studies other than the Rough Fire. Additionally, the geographic spread of smoke PM_2.5_ varies between data sets for each fire event with no consistent pattern.

## 4 Discussion

This is the first study to comprehensively compare multiple daily smoke PM_2.5_ data sets across several short‐ and long‐term metrics over a long‐term period (11 years). This analysis builds upon recent work, including one study comparing the performance of ML models of total PM_2.5_ during fire events (Considine et al., 2023) and another comparing CTM and ML smoke data sets in the high‐fire year of 2020 to evaluate implications for health impact assessment (Qiu et al., 2024). Understanding the accuracy of these smoke PM_2.5_ data sets—which cannot be directly measured using physical monitoring methods—is particularly important given the sparse coverage of U.S. EPA monitors, and disproportionate exposures to smoke for the population in regions outside the network (Zhang et al., 2023).

Scholars have identified several challenges in using ground station monitoring observations for modeling and validation, as monitors often have varying placement density, sampling schedules, and gaps in coverage in rural areas (Diao et al., 2019). Gaps in the regulatory monitoring network highlight the value of temporary monitors and low‐cost sensors for validating exposure surfaces (Considine et al., 2023; Diao et al., 2019). These spatial gaps are particularly important for public health researchers to consider as they utilize exposure surfaces for health impact assessments driven by either short‐ or long‐term exposure estimates in both rural and urban areas. To bridge gaps in monitoring networks, scholars have turned to various models such as those we compare in this study; understanding their differences and validity is therefore critical for ensuring their responsible deployment in epidemiological studies and health impact assessments.

We find considerable differences between the four data sets with respect to summary statistics and exposure metrics, as well as paired *t*‐tests comparing the aggregated metrics at the tract level. The Wilkins data set, using the U.S. EPA's CMAQ model, generally had the highest overall smoke concentrations, number of weeks with average concentration greater than 5 μg/m^3^, and most smoke waves. While the Wilkins data set appears anomalously high compared to the other three models in this study, a supplementary validation using USFS monitors deployed during several fires provides evidence that all four data sets may be underestimating smoke PM_2.5_ in certain cases, particularly extreme fire events. As noted previously, however, we can only draw cautious conclusions from this analysis considering the limitations associated with these monitors. This includes but is not limited to the monitors' measurement of only total PM_2.5_, not smoke PM_2.5_, which suggests some amount of underestimation is to be expected (though many fires do occur in more rural areas with fewer sources of non‐smoke PM_2.5_). Our findings do generally cohere with Considine et al. (2023) who evaluated ML data sets without a parsed smoke PM_2.5_ fraction during wildfire events, demonstrating underprediction of smoke PM_2.5_, particularly during high exposure events. Notably, existing monitoring evidence demonstrates that PM_2.5_ concentrations during large fires have historically reached exceptionally high levels: approximately 700 μg/m^3^ during one of California's 2020 fires (Navarro & Vaidyanathan, 2020) and up to 545 μg/m^3^ during the 2015 Rough Fire (Cisneros et al., 2021). Such extreme values have critical public health implications and may be underrepresented in modeled smoke PM_2.5_ data sets. In specific cases, however, the nearby population may not necessarily be exposed to such elevated concentrations due to evacuation or other behavioral adaptations (Burke et al., 2022). Regardless, these results suggest that the ML models and Zhang's ML‐based data fusion model may benefit from incorporating higher PM_2.5_ concentrations measured near fires, such as the USFS data set.

Our case study analysis shows nuance in the differences between the four data sets, with some data sets with similar data inputs and methods presenting seemingly incongruent estimates and trends varying by case study region and time period. Notably, a recent analysis compared the Zhang, Childs, and Aguilera data sets for 2010 and 2018 at the ZIP code level and found stronger agreement for 2018, with the authors suggesting that the models are more aligned in high fire years (Orr et al., 2025). While informative with respect to the inconsistencies between the data sets, our case studies did not offer an indication of specific regional or temporal trends, which makes it challenging to suggest drivers of variation between data sets. For example, while the ML data sets are highly correlated, the average estimates for Aguilera are consistently severalfold lower than Childs, as presented in the summary statistics (Figure 1) and case study analysis (Table 2).

There are several reasons that the ML outputs may differ, including the binary smoke day characterization. The Childs methodology included a process to supplement the HMS data by simulating air trajectories using the Hybrid Single‐Particle Lagrangian Integrated Trajectory (HYSPLIT) model as an attempt to alleviate limitations with cloud cover (Childs et al., 2022). This approach could potentially be driving some differences when compared to the Aguilera data set. In terms of spatial resolution, both approaches used population‐weighting to some extent, but the Childs approach used both area‐ and population‐weighting to assign concentrations to census tracts from their native 10‐km grid, while the Aguilera approach estimated concentrations directly at census tract population‐weighted centroids. The approaches differ in several other ways; Childs estimates smoke PM_2.5_ based on PM_2.5_ anomalies at U.S. EPA ground stations on the classified smoky days, subtracting from all‐source PM_2.5_ at monitoring stations and then using the smoke PM_2.5_ as inputs in their ML model, whereas Aguilera attempts to estimate daily background (non‐smoke) PM_2.5_ using random forest‐based imputation after estimating all‐source PM_2.5_ on all days and in all census tracts. These differences likely account for some of the discrepancies observed between both statistically derived data sets. With respect to statistical approaches, it is also important to note that by definition, it is difficult to extrapolate from training data, and these approaches may not be optimal to capture extreme values. However, overall, they are less susceptible to model choices and some random processes in dynamical approaches.

As mentioned previously, there is also considerable uncertainty resulting from the use of HMS plumes in the atmospheric column as a binary proxy of a day with ground‐level smoke, which is the approach used in the Childs and Aguilera ML data sets. This has been investigated in several studies, with one analysis focused on the relationship between various pollutants during western U.S. fire events finding that HMS plumes missed the identification of a specific smoke day with concentrations greater than 100 μg/m^3^ at certain time points (Buysse et al., 2019). Another recently published study evaluated the agreement in using HMS as an indicator of surface smoke presence, recommending researchers proceed with caution after discovering multiple biases in this approach, including an overestimation of smoke days and trends when using light HMS smoke plumes, which often correspond to very low levels of PM_2.5_ (T. Liu et al., 2024).

Ultimately, this analysis lends itself to several implications for researchers to consider when designing health impact assessment studies, as discussed in previous studies that explored choices of inputs such as dose‐response values and exposure surfaces (Cleland et al., 2021; Considine et al., 2023; Gan et al., 2017; Qiu et al., 2024). Considine et al. (2023) suggested that the use of ML modeled PM_2.5_ estimates in health impact analyses could result in exposure measurement error due to underprediction, based on comparisons to temporary monitors deployed during fires (Considine et al., 2023). Another recent study found that just one year of different exposures for three data sets over a 15‐year period influenced significant variation in the mortality exposure‐response function (Qiu et al., 2024). Until future research further refines exposure surface modeling to approach more of a gold‐standard for neighborhood‐level (i.e., census tract) exposure estimation, there may be added value in using multiple exposure surfaces for health impact analyses as a sensitivity. Ultimately, our results imply the potential for large error margins for any health impact assessment using these data sets, which has further downstream implications depending on how those findings are interpreted and applied.

Additionally, erroneously high concentrations in CTM data sets were also noted in Qiu et al. (2024) for the western U.S., despite using a different CTM model in addition to CMAQ. In our analysis, we see very high concentrations from CMAQ in the Wilkins data sets, particularly for the Wine Country Fires. Together with potential underestimation in the ML data sets, these uncertainties highlight the potential benefits of using binary, categorical, or binned smoke exposures in epidemiological analyses to avoid errors due to extreme concentration outliers (Considine et al., 2023), which has been done in various ways in recent studies (Qiu et al., 2024). Although researchers found considerable misclassification when using these methods to assign air quality index categories, these errors were reduced with a binary smoke versus non‐smoke classification (Considine et al., 2023).

This analysis has several limitations. These data sets are all developed and provided at different fundamental spatial resolutions, which makes comparing them challenging. The Wilkins and Childs data sets are less spatially resolved than the others. The Wilkins data was developed at the 12‐km scale, and the Childs data set at the 10‐km scale, and both were aggregated to the tract‐level (Wilkins to the population‐weighted census tract centroid, and Childs using area‐ and population‐weighting), which will inevitably affect precision, particularly taking into consideration sharp spatial gradients for events such as wildfires. Additionally, for the case studies, we included all census tracts in affected counties—in which the fire perimeters fell—in our assessment of exposure, with an underlying assumption that smoke has spread to these areas. To take a different approach, for example, to assign specific census tracts that fall within a given buffer of the fire within which we would expect smoke impacts, would introduce considerable uncertainty, as the pattern of smoke transport varies widely depending on season and meteorology. Therefore, we take a conservative approach, but note that exploring various methods of assigning impacted census tracts surrounding fires is an area for future study. In addition, smoke concentrations during the case study period may reflect contributions from fires other than the focal events, so while the internal comparison remains valid, the results should be interpreted in that context. Finally, our analysis is limited to California due to the availability of certain data and the intended focus on smaller regional case studies as illustrative examples, limiting the generalizability of our results. Such examples, however, and our overall comparison of the data sets, are particularly relevant to the western U.S. and other fire‐prone regions.

These results have important implications for epidemiological research. There is abundant literature assessing the health impacts of short‐term wildfire smoke exposure and emerging studies about long‐term wildfire smoke exposure on various outcomes including respiratory complications, stroke, and premature mortality (Connolly et al., 2024; Gould et al., 2024; Hao et al., 2026). Typically, epidemiological studies use only one wildfire smoke product, and all inference relies on the accuracy of this selected or available product. Here, we show how selected metrics (i.e., frequency, intensity, and duration) vary substantially across four distinct wildfire smoke products. Results from these studies are critical to inform prevention strategies, including early warning systems, and obtaining reliable estimates is essential. As described above, there is no gold standard for wildfire smoke exposure, and all products rely on different assumptions, variables, and processes. This suggests that future epidemiological studies should, when possible, use and compare multiple wildfire smoke products when estimating health impacts. Future research is thus also needed to better understand the drivers of these identified discrepancies across wildfire smoke products.

This exploratory study presents several opportunities for further work to elucidate the drivers of differences between these models and their implications for downstream health impact analyses. In their recent comparison of several data sets over 2 years, Orr et al. (2025) suggested that as smoke PM_2.5_ exposure models are developed and refined, further analysis could lead to insights on which specific features are driving differences in model outputs. Our findings demonstrating considerable differences between the four data sets suggest the potential utility of merging multiple exposure surface products to develop a calibrated surface (Gan et al., 2017; Qiu et al., 2024). Combining CTM and ML models provides an opportunity to blend the positive features of each modeling approach (Jaffe et al., 2020), including developing more spatially resolved exposure estimates without compromising the inclusion of simulated chemical and physical atmospheric processes. The Zhang data set included in our analysis combines CTM and ML approaches to develop a spatially resolved 1‐km surface and demonstrates promise in this regard (Zhang et al., 2023). In the recent study comparing CTM and ML data sets in 2020, findings demonstrated that a calibrated approach outperformed the CTM and ML models when validated with ground station monitors and low‐cost sensors, and the authors therefore recommend this exposure surface approach for downstream health impact assessment (Qiu et al., 2024). Our findings also largely suggest that regardless of which data set is utilized, systematic sensitivity analyses are warranted.

Another next step is extending Qiu et al.’s (2024) recent study comparing the health impact assessment implications of smoke database selection by leveraging this set of California models over the much longer 11 years they are available. Future research could also validate these data sets using strategically placed monitors and low‐cost sensors not used in model training, such as the PurpleAir network. Ultimately, it is crucial to develop targeted research approaches designed to quantify the predictive value added from supplementing traditional CTM modeling with ML approaches, including with respect to improving the spatial resolution of exposure surfaces past 12‐km to estimate exposures and downstream population health impacts at the neighborhood level.

## 5 Conclusions

This study is the first to comprehensively compare multiple daily smoke PM_2.5_ data sets developed using diverse methods over a long‐term period, highlighting substantial differences between the data sets using various metrics and validation. Our findings underscore the challenges of estimating smoke PM_2.5_ accurately, presenting potential estimation errors in both directions. These inconsistencies present an opportunity to focus development on calibrated surfaces that combine strengths of multiple exposure measurement methods. Future research should extend these comparisons to explore variations for health impact assessment and validate data sets with available monitoring networks. Given the devastating January 2025 wildfires in Los Angeles exposing much of the County's population of more than 10 million to elevated air pollution, this California‐specific analysis is particularly timely.

## Conflict of Interest

The authors declare no conflicts of interest relevant to this study.

## Supporting information

Supporting Information S1

## Data Availability

The data and code on which this article is based are available in (Connolly, 2026).
