# Supplementary material for: A Comparative Analysis of Wildland Fire Smoke PM2.5 Exposure Estimates Across California From 2008 to 2018
Source: Geohealth. 2026 Jun 17;10(6):e2025GH001575. doi: 10.1029/2025GH001575 (PMC13273639; doi:10.1029/2025GH001575)
Supplement: Supplementary file 1 — Supporting Information S1 [file GH2-10-e2025GH001575-s001.pdf]

*GeoHealth*

Supporting Information for

# A comparative analysis of wildland fire smoke $PM_{2.5}$ exposure estimates across California from 2008-2018

Rachel Connolly[1,2], Jenny T. Nguyen[3,4], Aron Walker[3], Joseph Wilkins[5], Yiqun Ma[4], Rosana Aguilera[4], Chen Chen[4], Alexander Gershunov[4], Joan A. Casey[6,7], Minghao Qiu[8], Danlu Zhang[9], Yang Liu[10], Tarik Benmarhnia[4], Michael Jerrett[1,3], Miriam E. Marlier[3]

[1] Center for Healthy Climate Solutions, Fielding School of Public Health, University of California Los Angeles, Los Angeles, CA, USA

[2] Luskin Center for Innovation, University of California Los Angeles, Los Angeles, CA, USA

[3] Department of Environmental Health Sciences, Fielding School of Public Health, University of California Los Angeles, Los Angeles, CA, USA

[4] Scripps Institution of Oceanography, University of California San Diego, La Jolla, CA, USA

[5] Department of Earth, Environment and Equity, Howard University, Washington, DC, USA

[6] Department of Environmental and Occupational Health, University of Washington School of Public Health, Seattle, WA, USA

[7] Department of Epidemiology, University of Washington School of Public Health, Seattle, WA, USA

[8] School of Marine and Atmospheric Sciences & Program in Public Health, Stony Brook University, Stony Brook, NY, USA

[9] Department of Biostatistics and Bioinformatics, Rollins School of Public Health, Emory University, Atlanta, GA, USA

[10] Gangarosa Department of Environmental Health, Rollins School of Public Health, Emory University, Atlanta, GA, USA

## Contents of this file

Text S1
Figures S1 to S10
Tables S1 to S2

## Introduction

This supplement contains supplemental methodology details on the calculation of long-term exposure metrics, as well as supplemental figures and tables.

**Text S1. Metric calculation details**

We applied four long-term smoke $PM_{2.5}$ metrics to compare the frequency, duration, and intensity of each dataset as discussed by Casey et al. (2024). This analysis was conducted at the census tract level.

The first metric is the number of weeks each year for which mean smoke $PM_{2.5}$ concentrations exceeded 5 μg/m$^3$ . This was calculated by delineating seven-day periods in each year starting January 1st, with any days beyond the 52nd week assigned to that week to avoid including particularly short weeks.

The second metric is the number of days each year for which smoke $PM_{2.5}$ concentrations were >1 μg/m$^3$. This was adapted from 0 μg/m$^3$ in the Casey et al. study. We made this adjustment because the Wilkins smoke $PM_{2.5}$ dataset was derived by subtracting CTM-modeled non-fire $PM_{2.5}$ from all-source $PM_{2.5}$. As a result of this methodological approach, many daily concentrations were near zero, but slightly above, so 1 μg/m$^3$ was more representative of whether or not a smoke day was present.

The third metric is the number of smoke waves each year, defined as instances of ≥2 consecutive days with >15 μg/m$^3$ smoke $PM_{2.5}$.

The fourth and final metric is the mean annual smoke $PM_{2.5}$ concentration in each census tract. We did not evaluate the mean daily smoke $PM_{2.5}$ concentration during the peak week of exposure (the fifth metric in Casey et al.) as there is no singular peak week for each census tract because concentrations vary between the four datasets.

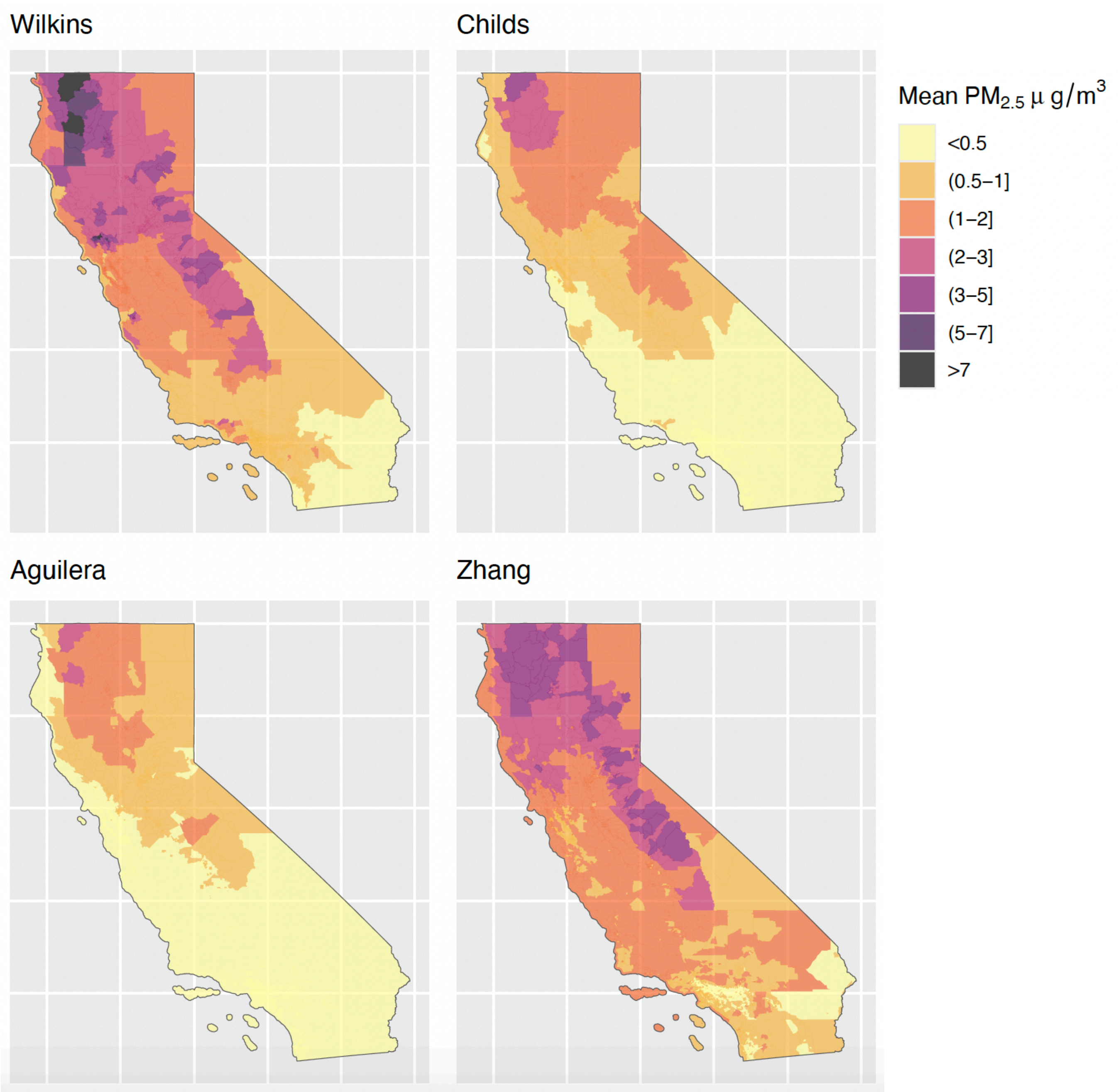


**Figure S1.** Average model-estimated smoke $PM_{2.5}$ over 2008-2018 for all four datasets at the census tract level

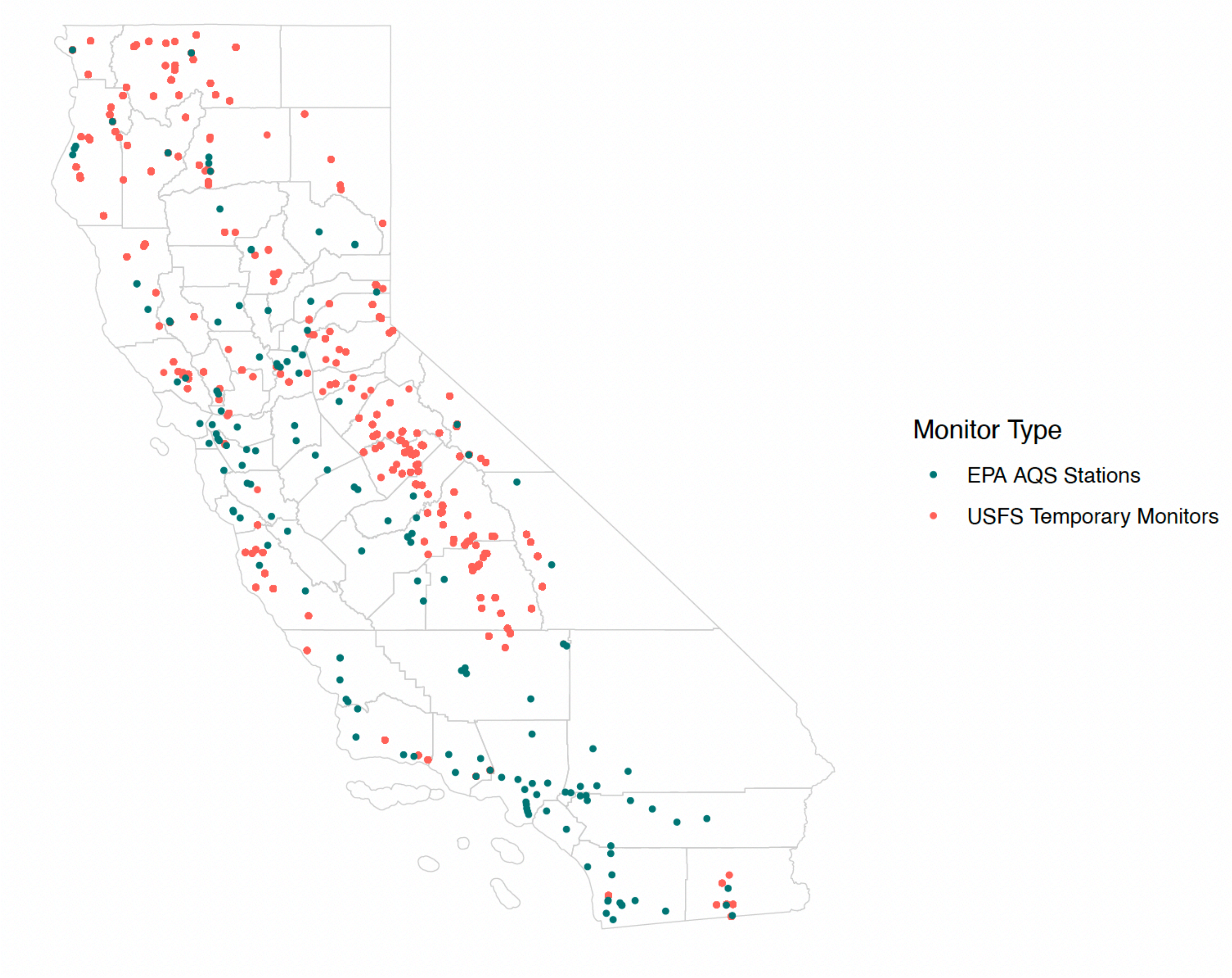


**Figure S2.** USFS temporary monitors alongside U.S. EPA Air Quality System (AQS) stations

Note: We include AQS stations that monitored $PM_{2.5}$ at some point from 2008-2018.

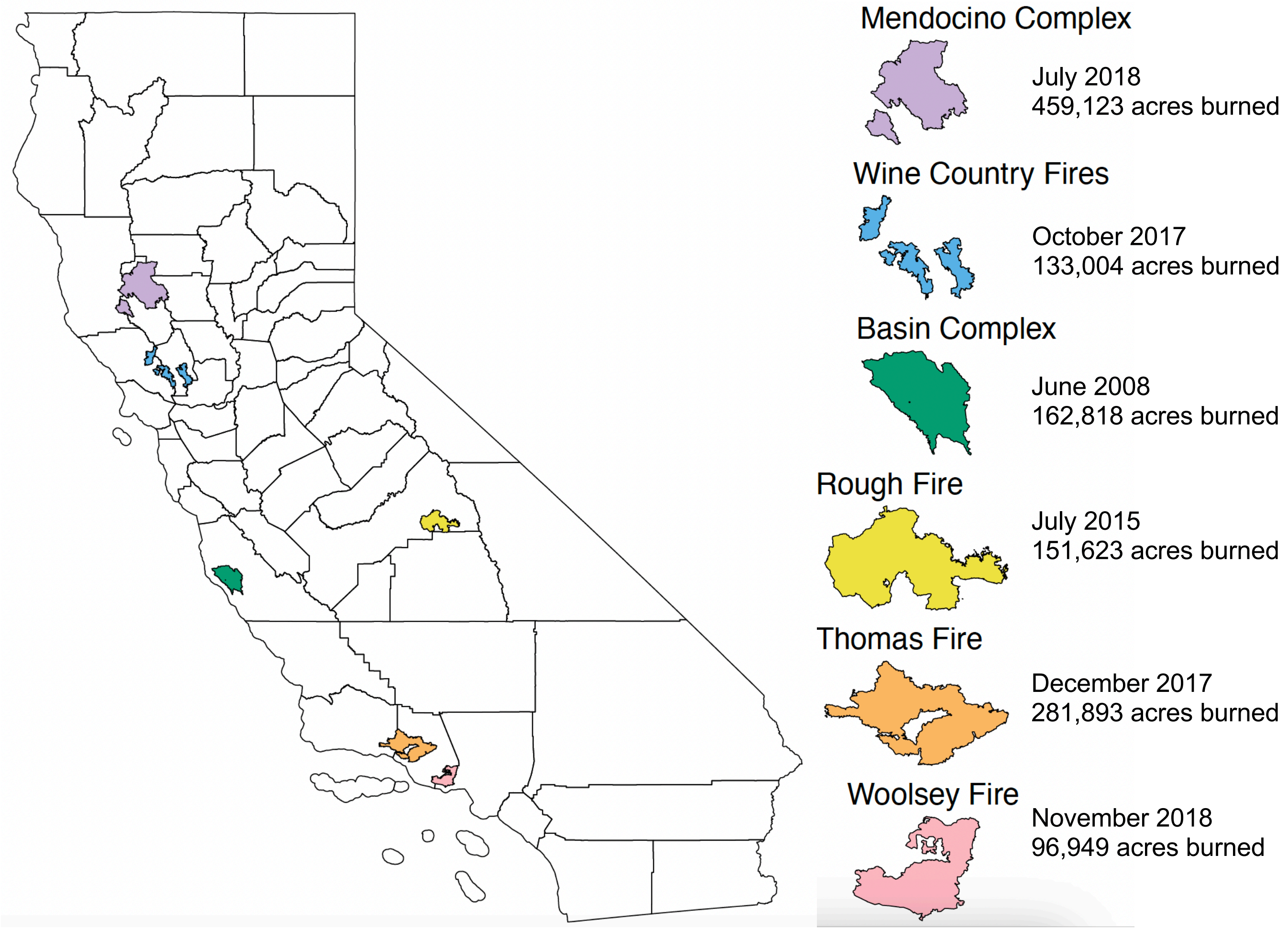


**Figure S3.** Fire perimeters, date, and total area burned for the six case studies evaluated across the four datasets.

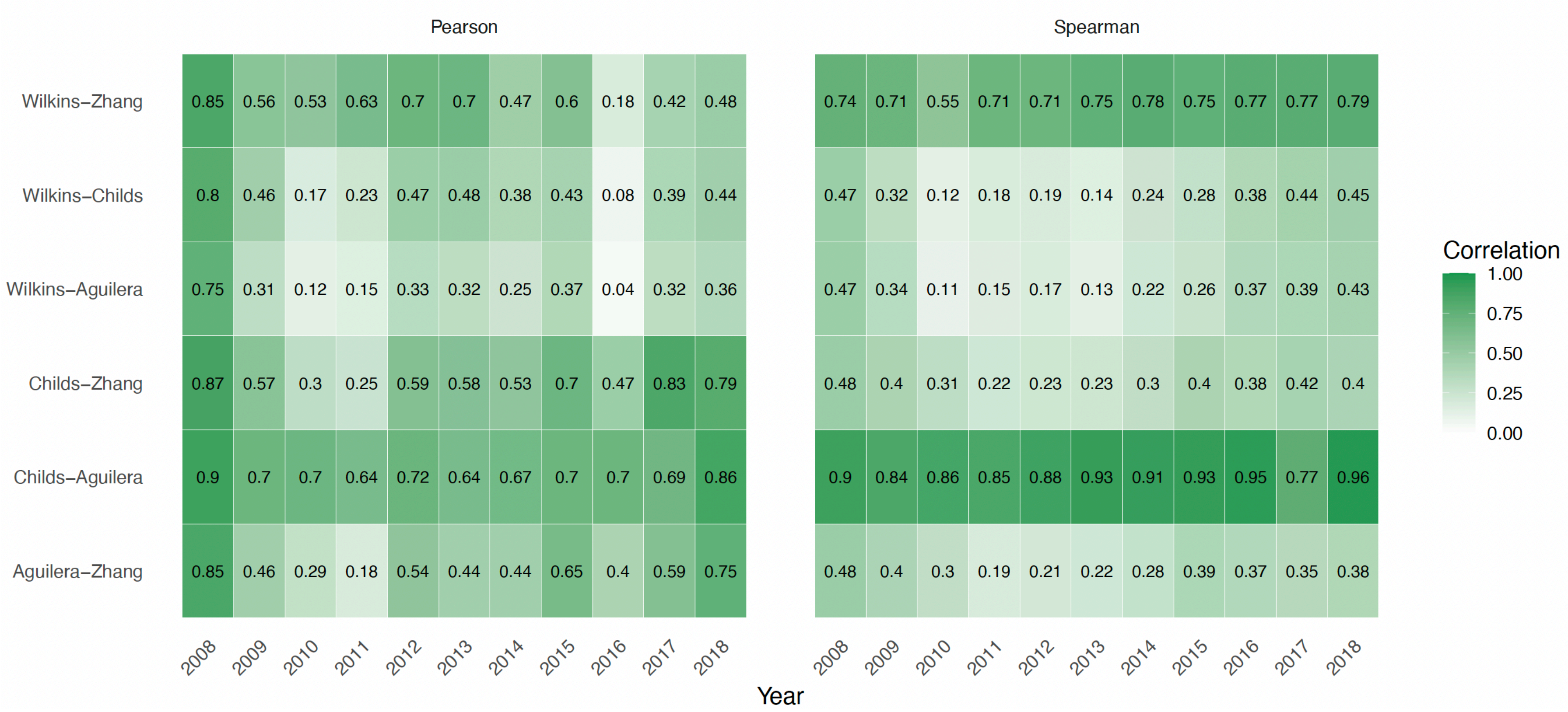


**Figure S4.** Pearson and Spearman correlations for daily estimates (tract-level), summer fire season only (June - Oct)

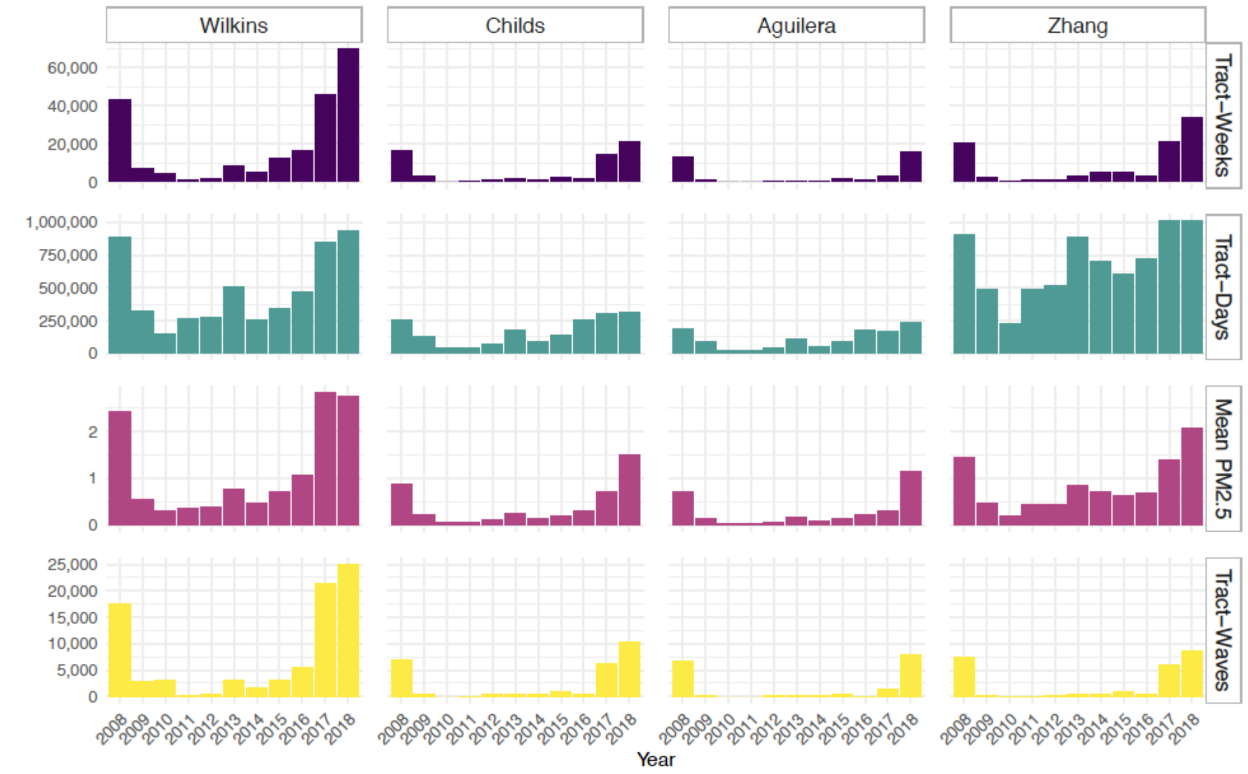


**Figure S5**. Temporal metrics of intensity, frequency and duration
Number of tract-weeks with smoke $PM_{2.5}$ exceeding 5 μg/m$^3$ (**first row**), number of tract-days with smoke $PM_{2.5}$ exceeding 1 μg/m$^3$, indicating nonzero smoke exposure (**second row**), mean smoke $PM_{2.5}$ in μg/m$^3$ (**third row**), number of tract-waves (**fourth row**)

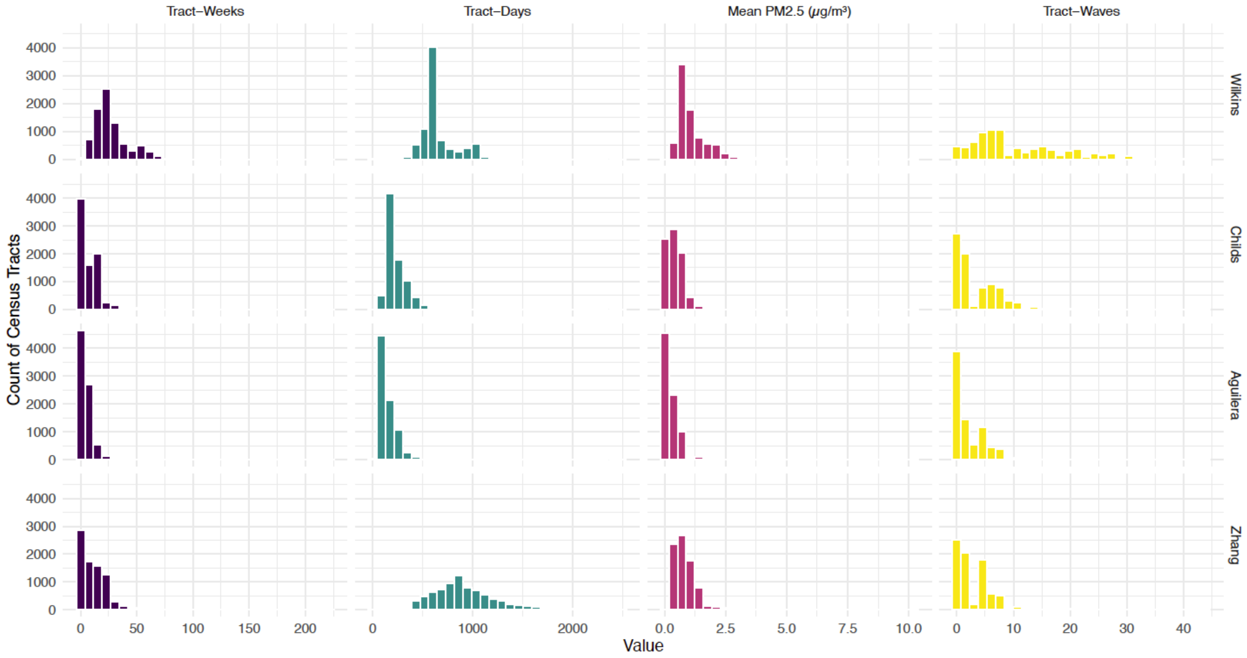


**Figure S6.** Histogram of tract counts for each metric (metrics shown by column, and datasets by row)

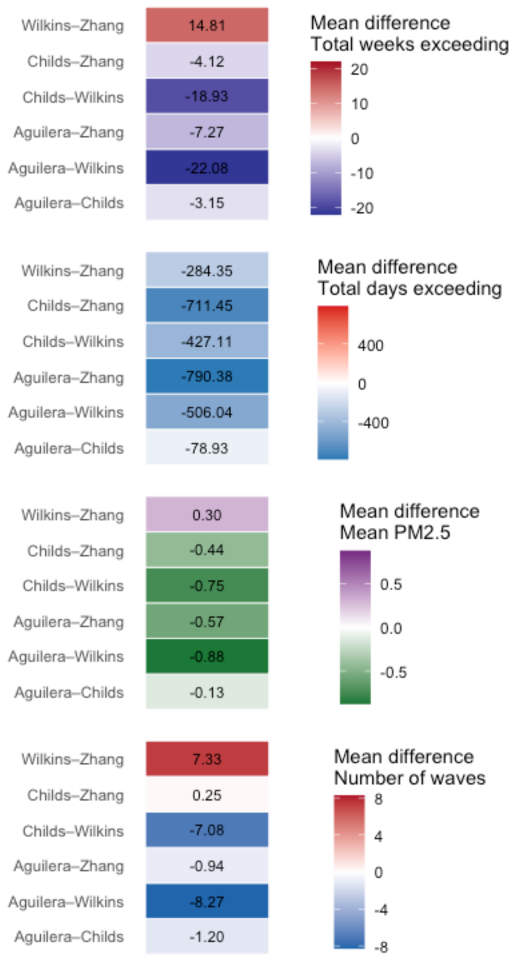


**Figure S7**. Mean differences for each metric calculated through the paired comparisons, each in its native unit and spanning 2008-2018: total weeks exceeding 5 μg/m$^3$, total days exceeding 1 μg/m$^3$, mean $PM_{2.5}$ in units of μg/m$^3$, and number of smoke waves.

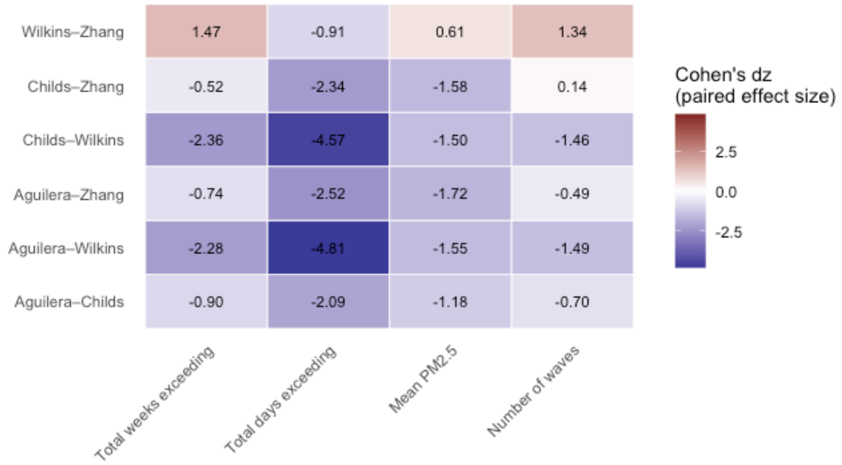


**Figure S8.** Cohen's d (effect size) for each metric and dataset as calculated through paired comparisons (t-test)

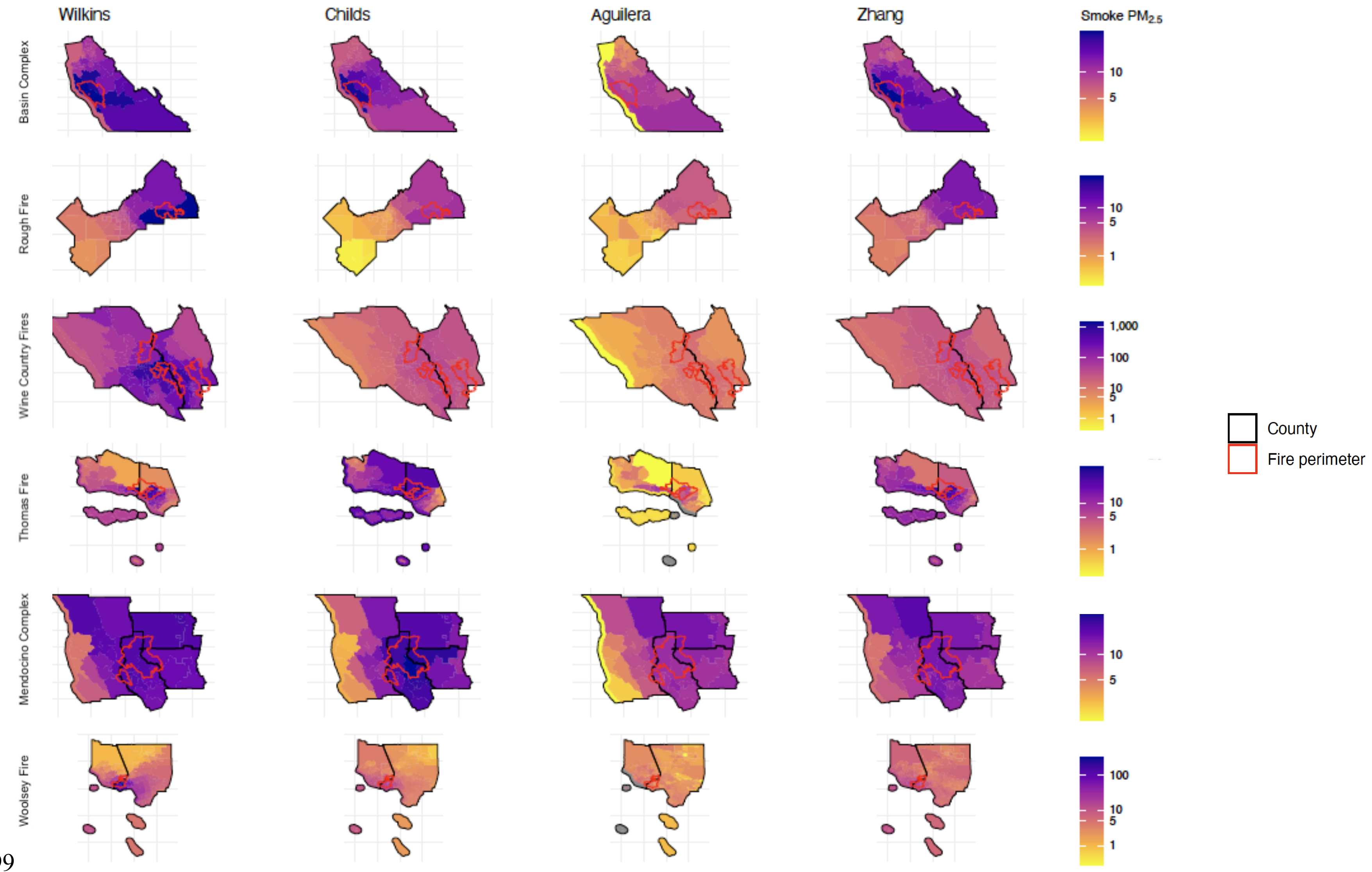


**Figure S9.** Mean smoke $PM_{2.5}$ concentrations during each case study (logarithmic scale, $\mu g/m^3$)

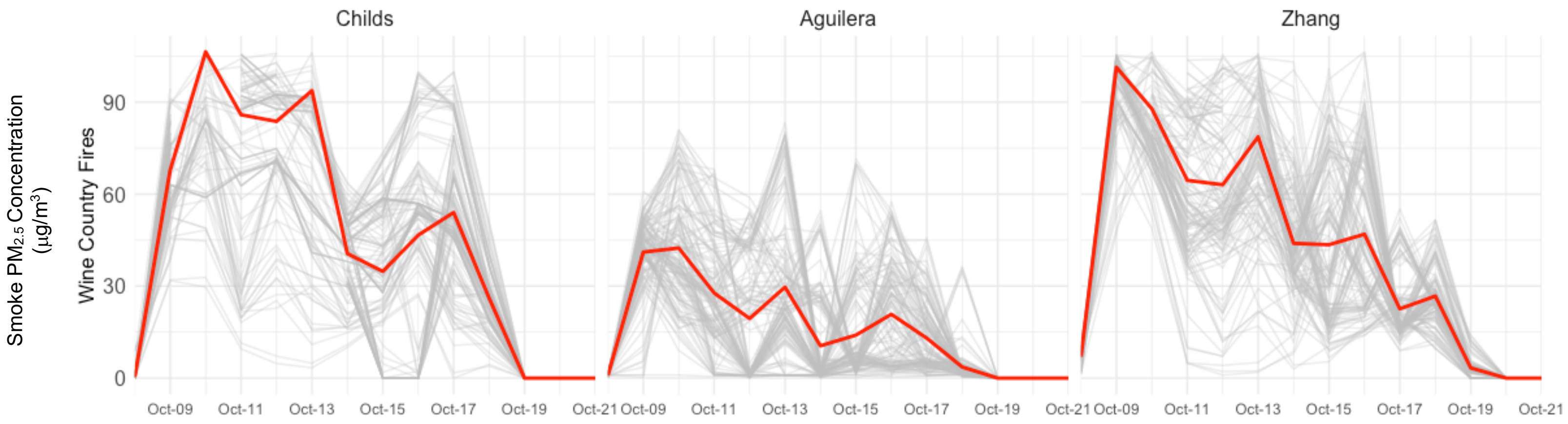


**Figure S10.** Average (red) and tract-level (gray) smoke $PM_{2.5}$ concentrations for the first two weeks of the Wine Country Fires for Childs, Aguilera, and Zhang datasets on an appropriate scale (also shown in Figure 3).

Notes: Gray lines reflect individual census tract estimates, and red lines the overall average for all tracts in all counties containing any portion of the fire perimeter. Extreme observations beyond each plot's y-axis are not included.

**Table S1**. Overview of datasets

| Name/author | Method | Available spatial resolution | Validation |
|---|---|---|---|
| Wilkins | Chemical transport model – CMAQ (BlueSky framework and SMARTFIRE2 for emissions) | 12-km grid | US EPA AQS ground station monitoring (2008-2012); AMET approach |
| Childs | Machine learning – gradient boosted trees | Census tract, ZIP code, 10-km | US EPA AQS ground station monitoring and PurpleAir network; 5-fold nested cross validation |
| Aguilera | Machine learning – random forest | Census tract, ZIP code | US EPA AQS ground station monitoring; 10-fold cross-validation and 5 hold-out sites |
| Zhang | Chemical transport model-based framework (CMAQ) with machine learning techniques for downscaling (within CMAQ, BlueSky framework and SMARTFIRE2 for emissions) | 1-km grid | US EPA AQS ground station monitoring; evaluated overall, temporal, and spatial 20-fold cross-validation, removed oversampled data |

Notes: AQS = Air Quality System; AMET = Atmospheric Model Evaluation Tool

**Table S2.** Model validation statistics compared to USFS temporary monitors (Note: comparing smoke $PM_{2.5}$ [modeled data] to total $PM_{2.5}$ [monitoring data])

| Year | # Paired obs. | Mean concentrations | | | | | Correlation coefficient (Pearson) | | | | $R^2$ | | | |
|---|---|---|---|---|---|---|---|---|---|---|---|---|---|---|
| | | ***USFS*** | Wilkins | Childs | Aguilera | Zhang | Wilkins | Childs | Aguilera | Zhang | Wilkins | Childs | Aguilera | Zhang |
| 2008 | 542 | 15.9 | 10.8 | 3.20 | 2.06 | 6.87 | 0.48 | 0.55 | 0.63 | 0.59 | 0.23 | 0.31 | 0.40 | 0.35 |
| 2009 | 736 | 13.6 | 1.65 | 0.90 | 0.63 | 1.96 | 0.29 | 0.36 | 0.25 | 0.32 | 0.08 | 0.13 | 0.06 | 0.1 |
| 2010 | 1,046 | 12.7 | 2.0 | 0.39 | 0.36 | 2.08 | 0.43 | 0.22 | 0.20 | 0.34 | 0.19 | 0.05 | 0.04 | 0.12 |
| 2011 | 1,022 | 9.90 | 2.17 | 0.57 | 0.62 | 2.58 | 0.23 | 0.27 | 0.21 | 0.28 | 0.05 | 0.07 | 0.04 | 0.08 |
| 2012 | 1,497 | 8.70 | 2.69 | 1.84 | 1.40 | 3.02 | 0.39 | 0.48 | 0.42 | 0.49 | 0.15 | 0.23 | 0.18 | 0.24 |
| 2013 | 1,381 | 14.6 | 8.36 | 5.09 | 2.70 | 5.75 | 0.54 | 0.62 | 0.36 | 0.61 | 0.29 | 0.39 | 0.13 | 0.37 |
| 2014 | 1,727 | 13.1 | 14.0 | 4.83 | 3.03 | 6.79 | 0.3 | 0.56 | 0.61 | 0.63 | 0.09 | 0.32 | 0.38 | 0.4 |
| 2015 | 1,819 | 18.2 | 13.9 | 4.90 | 3.39 | 7.76 | 0.34 | 0.50 | 0.38 | 0.48 | 0.12 | 0.25 | 0.14 | 0.23 |
| 2016 | 1,925 | 12.1 | 6.07 | 1.58 | 1.16 | 3.38 | 0.14 | 0.48 | 0.34 | 0.49 | 0.02 | 0.23 | 0.12 | 0.24 |
| 2017 | 2,988 | 17.3 | 13.2 | 5.92 | 1.96 | 6.87 | 0.28 | 0.78 | 0.66 | 0.76 | 0.08 | 0.60 | 0.44 | 0.57 |
| 2018 | 2,688 | 24.2 | 15.2 | 11.8 | 8.66 | 13.5 | 0.5 | 0.78 | 0.75 | 0.77 | 0.25 | 0.60 | 0.56 | 0.59 |

| Year | # Paired obs. | Root mean square error (RMSE) | | | | Mean bias (MB) | | | | Normalized mean bias (NMB) | | | |
|---|---|---|---|---|---|---|---|---|---|---|---|---|---|
| | | Wilkins | Childs | Aguilera | Zhang | Wilkins | Childs | Aguilera | Zhang | Wilkins | Childs | Aguilera | Zhang |
| 2008 | 542 | 22.1 | 16.2 | 16.8 | 14.7 | -5.09 | -12.7 | -13.9 | -9.05 | -32.0 | -79.9 | -87.1 | -56.9 |
| 2009 | 736 | 14.9 | 15.3 | 15.7 | 14.5 | -11.9 | -12.7 | -12.9 | -11.6 | -87.8 | -93.3 | -95.3 | -85.6 |
| 2010 | 1,046 | 14.8 | 16.6 | 16.6 | 15.1 | -10.7 | -12.3 | -12.3 | -10.6 | -84.3 | -96.9 | -97.1 | -83.7 |
| 2011 | 1,022 | 10.6 | 11.4 | 11.4 | 9.82 | -7.73 | -9.33 | -9.28 | -7.32 | -78.0 | -94.3 | -93.7 | -73.9 |
| 2012 | 1,497 | 10.0 | 9.93 | 10.4 | 8.95 | -6.01 | -6.85 | -7.29 | -5.68 | -69.1 | -78.8 | -83.9 | -65.3 |
| 2013 | 1,381 | 24.7 | 21.5 | 25.9 | 21.9 | -6.24 | -9.50 | -11.9 | -8.85 | -42.7 | -65.1 | -81.5 | -60.6 |
| 2014 | 1,727 | 65.3 | 18.2 | 18.6 | 16.5 | 0.89 | -8.26 | -10.1 | -6.3 | 6.78 | -63.1 | -76.8 | -48.2 |
| 2015 | 1,819 | 63.6 | 33.9 | 36.0 | 32.8 | -4.31 | -13.3 | -14.8 | -10.4 | -23.7 | -73.1 | -81.4 | -57.4 |
| 2016 | 1,925 | 57.1 | 17.5 | 18.4 | 16.2 | -6.03 | -10.5 | -10.9 | -8.72 | -49.8 | -87.0 | -90.4 | -72.1 |
| 2017 | 2,988 | 69.1 | 24.8 | 31.4 | 25.3 | -4.12 | -11.4 | -15.4 | -10.5 | -23.8 | -65.9 | -88.7 | -60.3 |
| 2018 | 2,688 | 38.4 | 30.8 | 34.2 | 31.0 | -8.95 | -12.4 | -15.5 | -10.6 | -37 | -51.2 | -64.2 | -44 |
